# Differential metabolic markers associated with primary open-angle glaucoma and cataract in human aqueous humor

**DOI:** 10.1186/s12886-020-01452-7

**Published:** 2020-05-06

**Authors:** Chen-Wei Pan, Chaofu Ke, Qin Chen, Yi-Jin Tao, Xu Zha, Yuan-Ping Zhang, Hua Zhong

**Affiliations:** 1 School of Public Health, Medical College of Soochow University, 199 Ren Ai Road, Suzhou, 215123 China; 2 Department of Ophthalmology, the First Affiliated Hospital with Nanjing Medical University, Nanjing, China; 3 Department of Ophthalmology, the First Affiliated Hospital of Kunming Medical University, No. 295 Xichang Road, Kunming, 650032 China; 4 Department of Ophthalmology, the Second Affiliated Hospital of Kunming Medical University, Kunming, China

**Keywords:** Open-angle glaucoma, Cataract, Epidemiology, Metabolomics

## Abstract

**Background:**

We aimed to identify metabolic biomarkers and investigate the metabolic alterations in relation to primary open-angle glaucoma (POAG) and cataract in human aqueous humor.

**Methods:**

Sixteen POAG patients undergoing surgical treatments and 24 patients undergoing cataract surgeries were included in this case-control study. We performed the metabolomic analysis of aqueous humor samples using a non-targeted gas chromatography coupled to time-of-flight mass spectrometer. The area under the receiver operating characteristic curve (AUC) was computed to assess the discrimination capacities of each metabolite marker. Databases including the Kyoto Encyclopedia of Genes and Genomes (KEGG) and MetaboAnalyst were utilized to search for the potential pathways of metabolites.

**Results:**

Aqueous humor metabolomic profiles could well distinguish POAG from controls. Fourteen metabolic biomarkers were identified as potential aqueous humor biomarkers for POAG, yielding AUC values from 0.62 to 0.86. In pathway analysis, Biotin metabolism was highly impacted, implying that these metabolic markers play important roles in the regulation of this pathway.

**Conclusions:**

This study identified valuable metabolic biomarkers and pathways that may facilitate an improved understanding of the POAG pathogenesis. The finding holds translational value in the development of new therapeutic measures for POAG.

## Background

Primary open-angle glaucoma (POAG) is the most common subtype of glaucoma and the major cause of irreversible blindness throughout the world [1]. Although numerous studies have identified several important ocular risk factors for POAG such as increased intraocular pressure (IOP) [2, 3], myopic refractive errors [4], larger optic disc size [5, 6] and thinner central corneal thickness [7, 8], these findings are limited in understanding the pathophysiology of POAG. Further knowledge regarding the pathophysiology might help to create new drug development research lines and expand current therapeutic targets for POAG. In current clinical practice, the treatment strategy of POAG mainly relies on IOP-lowering medications or surgeries. Although increased IOP is widely accepted to be the primary predictor for POAG, glaucomatous neuropathy is still observed in some patients with normal or even lower-than-normal IOPs, suggesting that other mechanisms exist in the pathophysiology of POAG.

Metabolomics is a widely used technology to assess biomarkers for diseases and provide molecular information regarding disease phenotype since metabolites are the ultimate product of gene, mRNA and protein activities [9]. Variations in the metabolome represent the interplay of genetic and environmental factors and are in relation to disease states, which may shed some lights in mechanism and pathophysiology of the disease [10]. With regard to eye diseases, metabolomics has been successfully used in identifying the metabolic signatures of diabetic retinopathy [11]. However, there were less studies focusing on POAG, especially in human participants. A previous analysis comparing plasma metabolic signatures as measured by mass spectrometry observed significant differences in some specific metabolic processes such as palmitoylcarnitine, sphingolipids, vitamin D-related compounds, and steroid precursors between POAG patients and healthy controls [12]. These differences observed in metabolome might be linked to mitochondrial dysfunction and energy metabolism changes [12]. However, we believe that aqueous humor samples are more sensitive to detect biomarkers in glaucomatous eyes, which may provide novel insights into more new pathogenic pathways for this ocular condition. To the best of our knowledge, few studies have focused on the aqueous humor metabolite markers of POAG in human beings. To address this gap, we performed a clinical-based case-control study and aimed to identify novel metabolite markers of POAG in human aqueous humor samples.

## Methods

### Study design and participants

A clinical-based case-control study was conducted on patients in two tertiary hospitals in China including the First Affiliated Hospital of Kunming Medical University and the First Affiliated Hospital with Nanjing Medical University. Two ophthalmologists (Qin Chen and Hua Zhong) collected aqueous humor samples during the surgical treatment in consecutive samples of 40 POAG and 40 cataract patients (20 cases and 20 controls in each hospital). Considering that no sample size calculation rationales are available for metabonomic study at current stage, the sample size was determined based on previous published literatures on metabonomic studies of aqueous humor samples as well as our available resources. In this study, cases were POAG participants who undertook surgical treatment and were free of cataract. POAG patients were considered to be free of cataract if the nuclear opalescence or nuclear color was less than 4, the cortical cataract score was less than 2, and the posterior subcapsular cataract score was less than 2 in the affected eye based on the Lens Opacities Classification System (LOCS) III [13]. Controls were participants who undertook cataract surgeries. The samples of aqueous humor (at least 20 μL) were extracted for each participant during the surgical treatment for both cases and controls. Aqueous humor samples were stored in a freezer at the temperatures of − 80 °C immediately after it was extracted during the surgical treatment.

The study was conducted following the tenets of the Helsinki Declaration and was approved by the Institutional Review Board of the Kunming Medical University. All patients included in the study provided written informed consent for aqueous humor samples to be extracted.

### Metabonomic profiling and data processing

First, derivatization of the samples was performed according to the protocols reported in a previous study [14]. Then, all samples were analyzed by gas chromatograph system coupled with a Pegasus HT time-of-flight mass spectrometer (GC-TOF-MS). GC-TOF-MS analysis was performed using an Agilent 7890 gas chromatograph system coupled with a Pegasus HT time-of-flight mass spectrometer. A 1 μL aliquot of the analyte was injected in a splitless mode. Helium was used as the carrier gas, the front inlet purge flow was 3 mL per minute, and the gas flow rate through the column was 1 mL per minute. The initial temperature was kept at 50 °C for 1 min, then raised to 310 °C at a rate of 20 °C per min and was kept for 6 min at 310 °C. The injection, transfer line, and ion source temperatures were 280 °C, 280 °C, and 250 °C, respectively.

Chroma TOF 4.3X software was used for raw peaks exacting, data baselines filtering and calibration of the baseline, peak alignment, deconvolution analysis, peak identification and integration of the peak area. Mass spectrum match and retention index match were considered in metabolites identification. We removed peaks detected in less than 50% of quality control (QC) samples or relative standard deviation (RSD) more than 30% in QC samples.

### Statistical analyses

First of all, peaks could be left through interquartile range denoising method. Then the missing values of raw data were filled up by half of the minimum value. A multivariate analysis was performed using the SIMCA14.1 software package (V14.1, Sartorius Stedim Data Analytics AB, Umea, Sweden). An unsupervised model of principal component analysis (PCA) with unit variance scaling was applied to show the distribution of origin data [15]. In order to obtain a higher level of group separation and get a better understanding of variables responsible for classification, supervised orthogonal projections to latent structures-discriminate analysis (OPLS-DA) were applied [16]. To refine this analysis, the first principal component of variable importance in the projection (VIP) was obtained. The VI*P* values exceeding 1 were first selected as changed metabolites. In addition, these selected metabolites were further validated at a critical *P* value of 0.05 using two sided student’s t-test. The area under the receiver operating characteristic curve (AUC) was computed to assess the discrimination capacities of each metabolite marker. Databases including the Kyoto Encyclopedia of Genes and Genomes (KEGG) [17] (http://www.genome.jp/kegg/) and MetaboAnalyst [18](http://www.metaboanalyst.ca/) were utilized to search for the pathways of metabolites.

## Results

### Characteristics of the participants

In the end of the study, 16 patients with POAG (40%) undergoing surgical treatments and 24 patients (60%) undergoing cataract surgeries from the two hospitals provided written informed consent for their aqueous humor samples to be taken for research purpose. Thus, the ratio for cases vs. controls was 1:1.5. The mean age was 72.5 years in participants with POAG (cases) and 74.2 years in participants with cataract (controls) and the difference was not statistically significant (*P* = 0.13). Women accounted for 54% of the study sample in cases and 62% in controls. No preoperative medications were used for all participants.

### Aqueous humor metabolic profiles

**Fig. 1:**
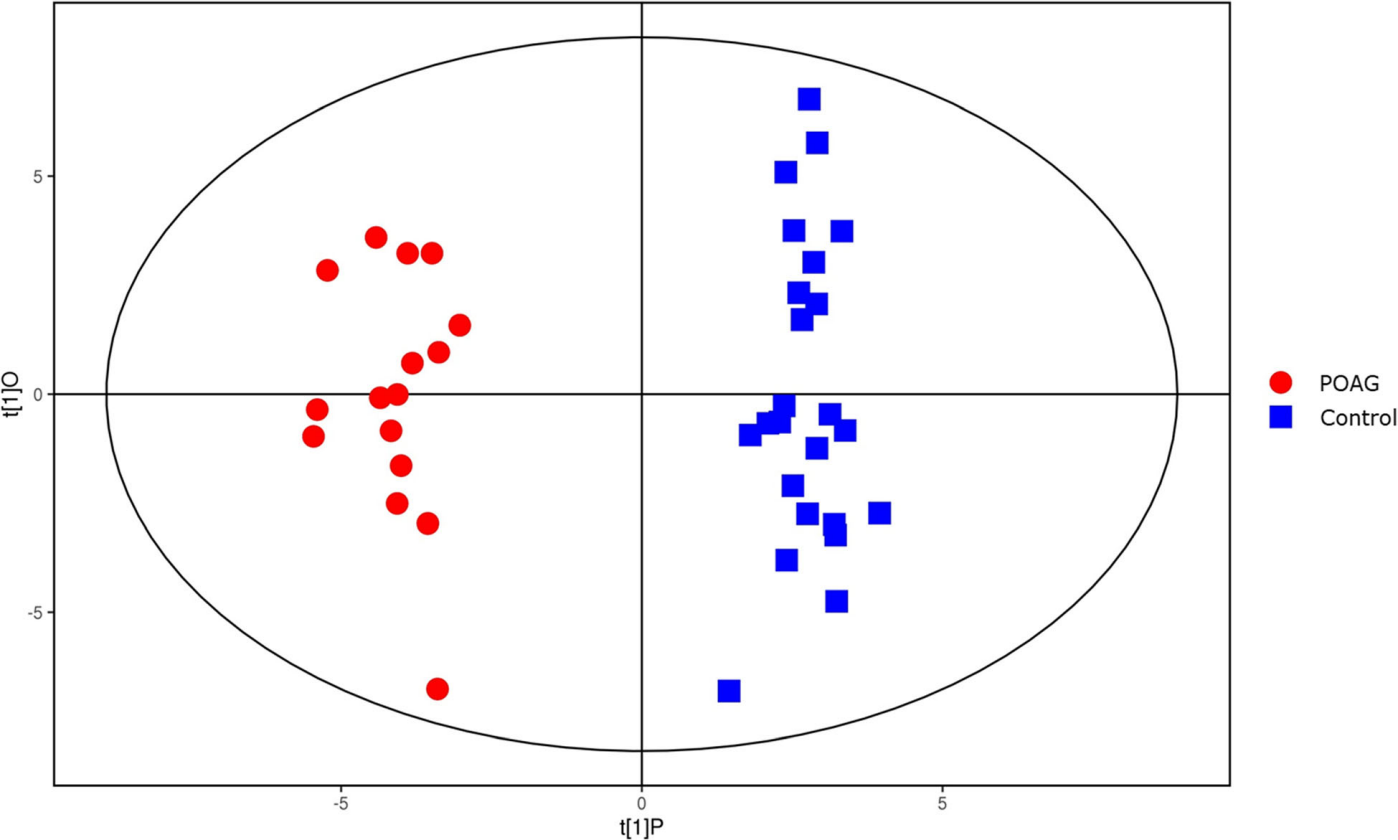
PLS-DA score plots for discriminating primary open-angle glaucoma and controls

**Fig. 2:**
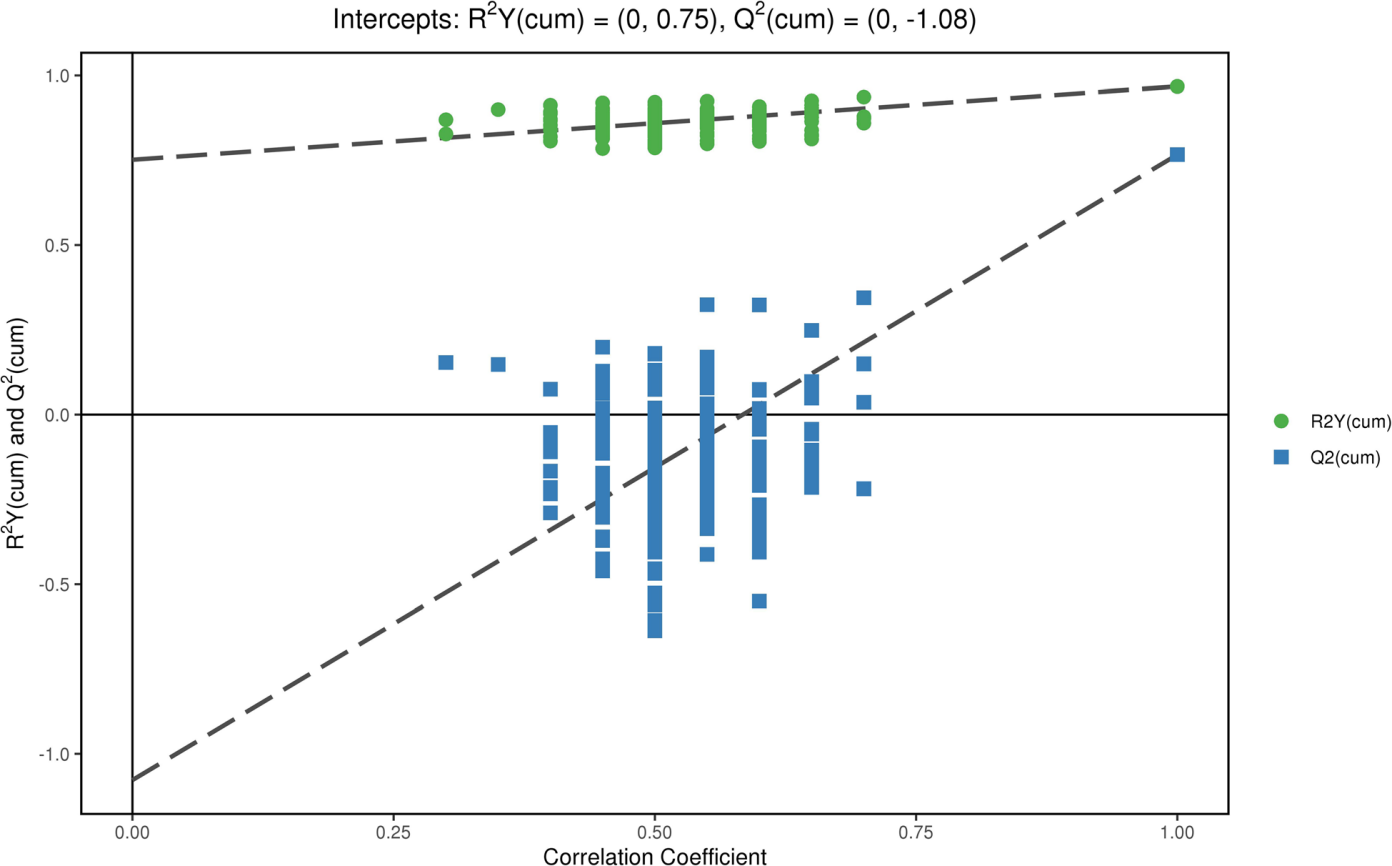
Validation plots for the OPLS-DA model

The PCA score plot showed that the QC samples were tightly clustered, supporting the robustness of the metabolic profiling platform (data not shown). The supervised OPLS-DA model was established to understand the holistic metabolic differences between POAG patients and controls. As shown in the OPLS-DA score plot, excellent separation between POAG patients and controls could be achieved (Fig. 1). The validation plot strongly supported the validity of the model, as all permuted R2 and Q2 values on the left were lower than the original points on the right (Fig. 2).

### Identification of potential biomarkers

**Fig. 3:**
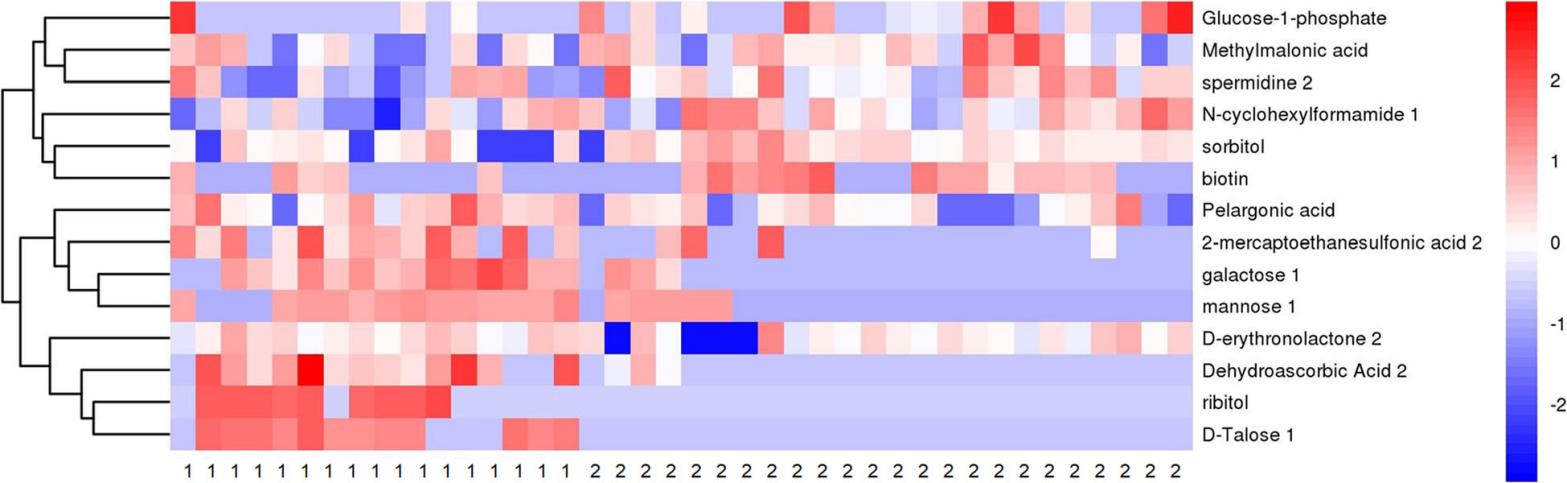
A heatmap showing the concentrations of 14 metabolite markers between primary open-angle glaucoma and controls (1: primary open-angle glaucoma, 2: controls)

Following the successful establishment of the OPLS-DA model, potential metabolic biomarkers were selected using the criteria of VIP or more than 1.0 and *P* value of less than 0.05. Finally, 14 metabolites were successfully selected and identified as potential biomarkers of POAG compared with controls (Table 1). Compared with controls, 6 metabolites were found to be decreased in those with POAG compared with cataract, including Biotin, Glucose-1-phosphate, Methylmalonic acid, N-cyclohexylformamide 1, Sorbitol, and Spermidine 2. In contrast, 8 metabolites, including 2-mercaptoethanesulfonic acid 2, D-erythronolactone 2, D-Talose 1, Dehydroascorbic Acid 2, Galactose 1, Mannose 1, Pelargonic acid and Ribitol, were found to be increased in participants with POAG compared with controls (Fig. 3). Those metabolite markers showed the potential to discriminate between POAG and controls, with AUC values ranging from 0.62 to 0.86 (Table 1).

**Table 1:** Potential metabolic biomarkers identified for primary open-angle glaucoma in aqueous humor samples

ID	Metabolite	VIP	*P* value	Up/Down Regulation	AUC
1	Glucose-1-phosphate	1.57	0.04	Down	0.64
2	Methylmalonic acid	1.16	0.04	Down	0.68
3	Spermidine 2	1.43	0.19	Down	0.69
4	N-cyclohexylformamide 1	1.57	0.01	Down	0.73
5	Sorbitol	1.67	0.02	Down	0.74
6	Biotin	1.13	0.05	Down	0.67
7	Pelargonic acid	1.02	0.01	Up	0.75
8	2-mercaptoethanesulfonic acid 2	2.67	< 0.001	Up	0.83
9	Galactose 1	3.01	< 0.001	Up	0.86
10	Mannose 1	2.43	< 0.001	Up	0.80
11	D-erythronolactone 2	1.27	0.05	Up	0.62
12	Dehydroascorbic Acid 2	2.77	< 0.001	Up	0.86
13	Ribitol	2.49	< 0.001	Up	0.78
14	D-Talose 1	3.26	< 0.001	Up	0.85

*VIP* Variable importance in the projection; *AUC* Area under the receiver operating characteristic curve

### Pathway analysis for potential biomarkers

**Fig. 4:**
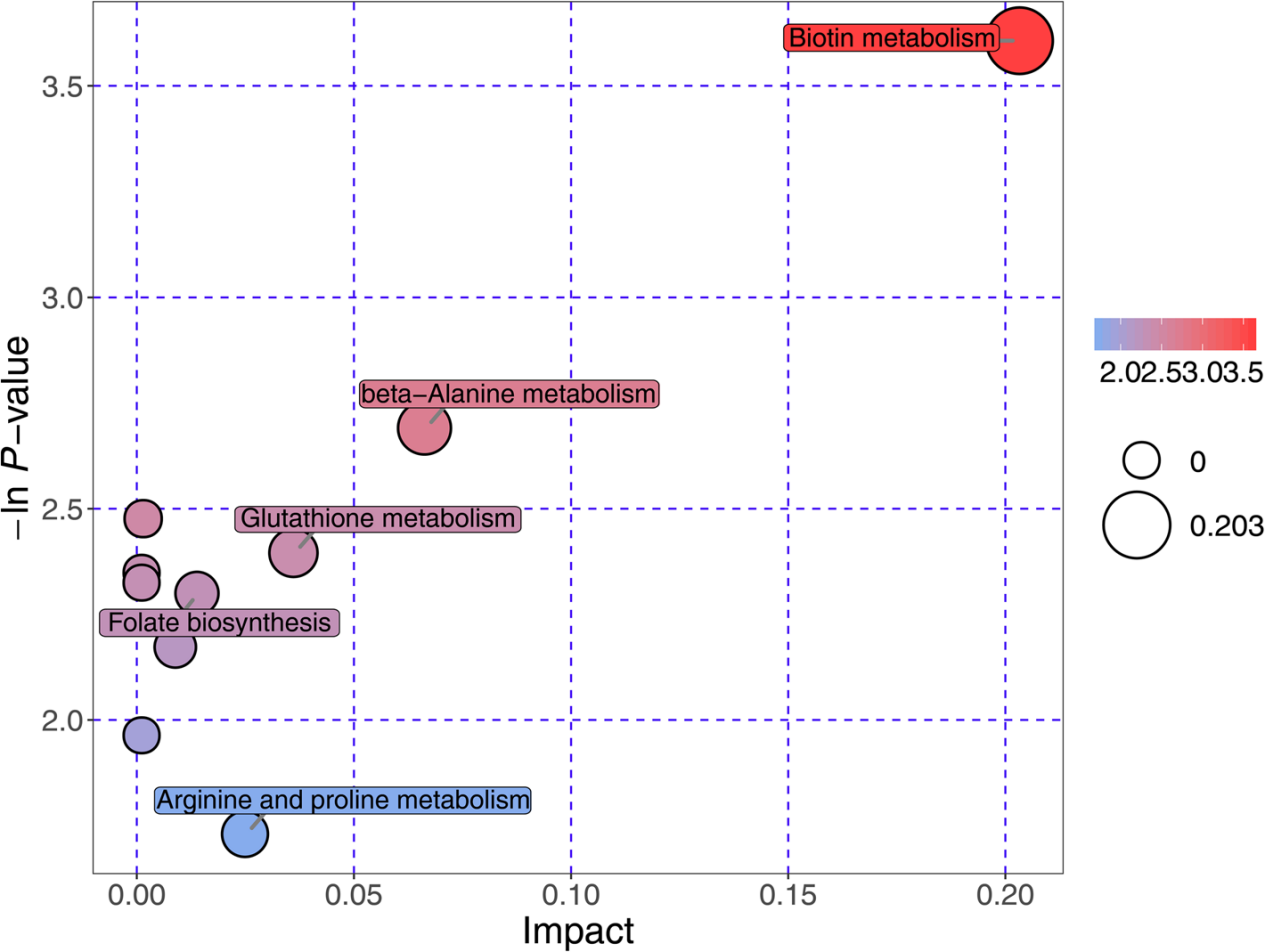
Enrichment analysis and pathway topology analysis for potential metabolic biomarkers of primary open-angle glaucoma

Pathway analysis, including enrichment analysis and pathway topology analysis, was further performed to understand the metabolic pathways that these potential biomarkers are involved in. A total of 5 pathways were significantly enriched at the significance level of 0.10, namely Biotin metabolism; Beta-Alanine metabolism; Glutathione metabolism; Folate biosynthesis; and Arginine and Proline metabolism (Fig. 4). Especially, Biotin metabolism was highly impacted, implying that these metabolic markers play important roles in the regulation of this pathway.

## Discussion

In clinical practice, POAG remains a poorly understood condition with limited therapeutic options. Therefore, there is a pressing need to develop personalized approach to guide clinical management. In this study, we systematically explored the metabolic differences in aqueous humor samples between patients with POAG and controls of patients with cataract. We identified fourteen metabolites as possible biomarkers in aqueous humor samples that had the potentials to distinguish POAG patients from controls. The identification of novel metabolite markers for POAG in human aqueous humor provides insights into potential new pathogenic pathways for this vision-threatening condition and could potentially lead to new drug development research lines.

Previous animal and human studies have provided some preliminary results on metabolites associated with glaucoma. For example, an experimental study found that metabolites in relation to osmotic stress, oxidative stress and glucose metabolism were related to the retinal ganglion cell death in rats, which is regarded as the common mechanism for glaucoma [19]. Another mouse model of chronic glaucoma identified unique profiles of sphingolipid and ceramide species between normotensive and hypertensive aqueous humor and found that sphingosine- and sphinganine-1-phosphates greatly increased in hypertensive mice [20]. With regard to the studies on humans, an untargeted plasma metabolomic study observed increased levels of palmitoylcarnitine; putatively-identified sphingosine, sphinganine, and decreased levels of sphingosine-1-phosphate in patients with POAG [12]. To the best of our knowledge, few studies have explored the POAG-associated metabolites in human aqueous humor samples. The analysis of metabolites in human aqueous humor may reveal novel potential biomarkers of POAG with greater sensitivity and specificity which might be detected in blood samples. We identified 14 metabolite markers in human aqueous humor which might potentially distinguish POAG from cataract. We also searched public databases such as KEGG [17] and MetaboAnalyst [18] and other published literatures, trying to find possible metabolic pathways for POAG observed in this study.

We found that Biotin metabolism was highly impacted in the pathway analysis, implying that Biotin may play major roles in POAG pathophysiology. Biotin was reported be oxidized into the retina [21]. It was also indicated that in vivo administration of biotin to early embryonic chick eyes at moderately elevated levels induced malformations, mainly affecting lens structures [22]. As the controls in this study were patients undergoing cataract surgeries whose lens structures might have been malformed compared with cases free of cataract, it is reasonable that the levels of Biotin is lowers in POAG patients observed in this study.

The phenomenon that Methylmalonic acid was reduced in POAG patients compared with controls probably suggested increased oxidative damage may play a major pathophysiological role in POAG [23].. Methylmalonic acid was reported to be able to provoke oxidative damage and compromise antioxidant defenses in nerve terminal and striatum [24]. According to the results of a recent meta-analysis including 22 case-control studies with 1614 with glaucoma and 1319 healthy controls, some biomarkers increased in glaucomatous aqueous humor such as superoxide dismutase and glutathione peroxidase. In addition, high levels of Methylmalonic acid were also found to increase the risk of optic neuropathy [25–27].

The level of Ribitol in aqueous humor was found to be higher in POAG compared with cataract in our study. Increased level of Ribitol was reported to play a role in prevention of cataract formation and act as a cofactor for glutathione reductase, which is linked to cataract formation by decreased glutathione levels in the lenses. On the other hand, Ribitol deficiency is implicated in the formation of cataracts due to the concentration of reduced glutathione in the lens and its ability to protect the tissue from oxidative damage [28]. Thus, our finding is consistent with previous studies.

The level of Sorbitol was expected to be lower in POAG patients as compared with those with cataract. It is well established that diabetes is a major risk factor cataract [29]. Sorbitol is a key marker in osmotic stress. Lens fiber cell resulting from excessive accumulation of sorbitol has been proposed as a possible mechanism in cataract formation in diabetic patients. A previous study reported a strong relationship between the abundance of polyol pathway metabolites sorbitol and blood glucose levels in lenses extracted from diabetic patients [30].

Mannose was found to be increased in POAG patients compared with cataracts. Mannose is a simple sugar but have a complex health effect on different parts of the body including the eyes. A previous study have indicated that an upregulation of the lectin pathway-associated mannose-serine-protease-2 was observed in the optic nerves of the optic nerve homogenate antigen group [31], which supports the finding in the current study.

We have to acknowledge some limitations of this study. One major limitation was the small sample size, which may have prevented changes in certain metabolites from being apparent. In addition, some sources of bias such as differences in the time of day of sample collection may have distorted the findings. Particularly, intake of drugs such as IOP-lowering medications in patients with POAG may have potentially altered the metabolome distributions. However, it is unlikely to control the effect of IOP-lowering medications between cases and controls in this study. Furthermore, the control group was patients undergoing cataract surgery rather than “healthy” individuals, and the selection of control group might have distorted the findings as cataract should have its own distinct metabolic profiling which should be different from POAG. It is ideal if we could obtain aqueous humor samples in healthy people without eye diseases. Finally, metabolic pathways of some metabolites identified in this study could not be founded in public databases or published literatures. Whether they are true biomarkers for POAG warrants further clarifications.

## Conclusions

This study investigated the metabolic markers associated with POAG in human aqueous humor samples. As a result, 14 metabolites were identified as potential biomarkers that could discriminate between patients with POAG and controls. Biotin metabolism was highly impacted in pathway analysis and may play important roles in the regulation of this pathway. Overall, this study provided new clues in the disease pathogenesis of POAG. Validation of the results is warranted in other studies.

## Data Availability

The datasets analyzed in this study are available from the corresponding author (Chen-Wei Pan, pcwonly@gmail.com) upon reasonable request.

## Abbreviations

POAG: Primary open-angle glaucoma
IOP: Intraocular pressure
LOSC: Lens opacities classification system
QC: Quality control
RSD: Relative standard deviation
PCA: Principal component analysis
VIP: Variable importance in the projection
AUC: Receiver operating characteristic curve
